# Comparing the Influence of Wildfire and Prescribed Burns on Watershed Nitrogen Biogeochemistry Using ^15^N Natural Abundance in Terrestrial and Aquatic Ecosystem Components

**DOI:** 10.1371/journal.pone.0119560

**Published:** 2015-04-17

**Authors:** Kirsten Stephan, Kathleen L. Kavanagh, Akihiro Koyama

**Affiliations:** 1 Forest, Rangeland, and Fire Sciences, University of Idaho, Moscow, ID, United States of America; 2 Department of Life and Physical Sciences and Cooperative Research, Lincoln University, Jefferson City, Missouri, United States of America; University of Waterloo, CANADA

## Abstract

We evaluated differences in the effects of three low-severity spring prescribed burns and four wildfires on nitrogen (N) biogeochemistry in Rocky Mountain headwater watersheds. We compared paired (burned/unburned) watersheds of four wildfires and three spring prescribed burns for three growing seasons post-fire. To better understand fire effects on the entire watershed ecosystem, we measured N concentrations and δ^15^N in both the terrestrial and aquatic ecosystems components, i.e., soil, understory plants in upland and riparian areas, streamwater, and in-stream moss. In addition, we measured nitrate reductase activity in foliage of *Spiraea betulifolia*, a dominant understory species. We found increases of δ^15^N and N concentrations in both terrestrial and aquatic ecosystem N pools after wildfire, but responses were limited to terrestrial N pools after prescribed burns indicating that N transfer from terrestrial to aquatic ecosystem components did not occur in low-severity prescribed burns. Foliar δ^15^N differed between wildfire and prescribed burn sites; the δ^15^N of foliage of upland plants was enriched by 2.9 ‰ (difference between burned and unburned watersheds) in the first two years after wildfire, but only 1.3 ‰ after prescribed burns. In-stream moss δ^15^N in wildfire-burned watersheds was enriched by 1.3 ‰, but there was no response by moss in prescription-burned watersheds, mirroring patterns of streamwater nitrate concentrations. *S*. *betulifolia* showed significantly higher nitrate reductase activity two years after wildfires relative to corresponding unburned watersheds, but no such difference was found after prescribed burns. These responses are consistent with less altered N biogeochemistry after prescribed burns relative to wildfire. We concluded that δ^15^N values in terrestrial and aquatic plants and streamwater nitrate concentrations after fire can be useful indicators of the magnitude and duration of fire effects and the fate of post-fire available N.

## Introduction

Fire is an integral component of ecosystem nitrogen (N) biogeochemistry in coniferous ecosystems of the Rocky Mountains, USA. Through combustion, a net loss of N contained in live and dead aboveground organic matter and forest floor occurs [1,2], followed by a range of post-fire alterations in N cycling associated with changes in plant cover, microbial activity, microclimate, and soil chemical environment. Despite the potentially large net loss of N through combustion [3], short-term increases in inorganic N in the soil are commonly observed [4–6]. This N can either be retained in recovering plant and microbial biomass, or leached into deeper soil layers and eventually into streams where it, in turn, contributes to aquatic N cycling [6]. Even though patterns of N distribution (N types and their concentrations) in recently burned ecosystems are commonly described [2,4,7], mechanisms behind the N dynamics leading to these observed patterns post-fire are not well understood. This is in part caused by the complexity of the N cycle and substantial small-scale spatial variation of microbial abundance and activity after fire [8].

The ratio of N stable isotopes (i.e., ^14^N and ^15^N) can be a useful tool to investigate ecosystem N dynamics. For instance, ^15^N isotope tracers were used to quantify gross N transformation rates in soil [5,9–11], and to identify NH_4_^+^ and NO_3_^-^ sinks and N cycling rates in terrestrial [12–14] and aquatic ecosystems [15,16]. Ratios of N stable isotopes at natural abundance, however, are more challenging to interpret because they integrate all N sources and processes concerning ecosystem components of interest [17–19]. Another challenge is the accurate measurement of δ^15^N values at natural abundance of small and variable pools of inorganic N in soil [19,20]. Despite such challenges, natural abundances of N stable isotopes have been used successfully as indicator of a specific ecosystem process if the process is dominant over others. In addition, studying N stable isotopes at natural abundance offers the advantage of obtaining insights into N dynamics without disturbing them [17] and have lower costs compared to the use of ^15^N tracers.

Recent studies have explored changes in N cycling after ecosystem perturbation using N stable isotopes at natural abundance. After clear-cutting, for example, foliar ^15^N enrichment has often been observed [21–23] and has been linked to uptake of enriched residual soil inorganic N caused by leaching losses of NO_3_^-^ depleted in ^15^N. The use of N stable isotopes in studying post-fire N cycling, however, has been sparse. In notable exceptions, Grogan et al. [24] found significant enrichment of foliage on burned sites that was attributed to post-fire reliance on NH_4_^+^ generated from enriched soil organic matter, Beghin et al. [25] studied δ^15^N in *Pinus sylvestris* tree rings before and after a stand-replacing fire, and Dunnette et al. [26] demonstrated that increased δ^15^N of lake sediment was associated with high-severity wildfires. From these studies, it is apparent that, in order to interpret plant ^15^N values, it is important to determine the form of N taken up by the plants. In the field, nitrate reductase activity (NRA) is a good indicator for NO_3_^-^ uptake and use by plant roots and foliage [27]. This method relies on native soil-NO_3_^-^, whereas studies on plant uptake of NH_4_^+^ or organic N require adding ^15^N tracer to the soil. In soils without external N added, however, it may be possible to use the fire-generated ^15^N signal in residual soil to trace the impact of fires to aquatic systems [28].

In this study, we explored use of N stable isotopes at natural abundance, N concentrations, and plant foliar NRA to gain insights into N biogeochemistry and terrestrial-aquatic linkages in small watersheds following forest fires of different fire severities. Specifically, we quantified δ^15^N values as well as N concentrations of several terrestrial and aquatic ecosystem components affected by moderate severity wildfires and low severity spring prescribed burns. The ecosystem components studied included soil, understory plants in upland and riparian areas (as important post-fire terrestrial N sinks), streamwater, and in-stream moss (as important post-fire aquatic N sink). By combining the results of foliar NRA, δ^15^N measurements, and N concentrations (the latter published in a companion study [6]) and by integrating the terrestrial and aquatic components of watershed ecosystems, we hoped to gain deeper insights into post-fire N biogeochemistry in headwater areas. Our objectives were to compare the magnitude of the post-fire response between wildfires and prescribed burns in specific ecosystem components and to quantify terrestrial-aquatic linkages after fires of different severity.

## Methods

### Study Sites

**Fig 1:**
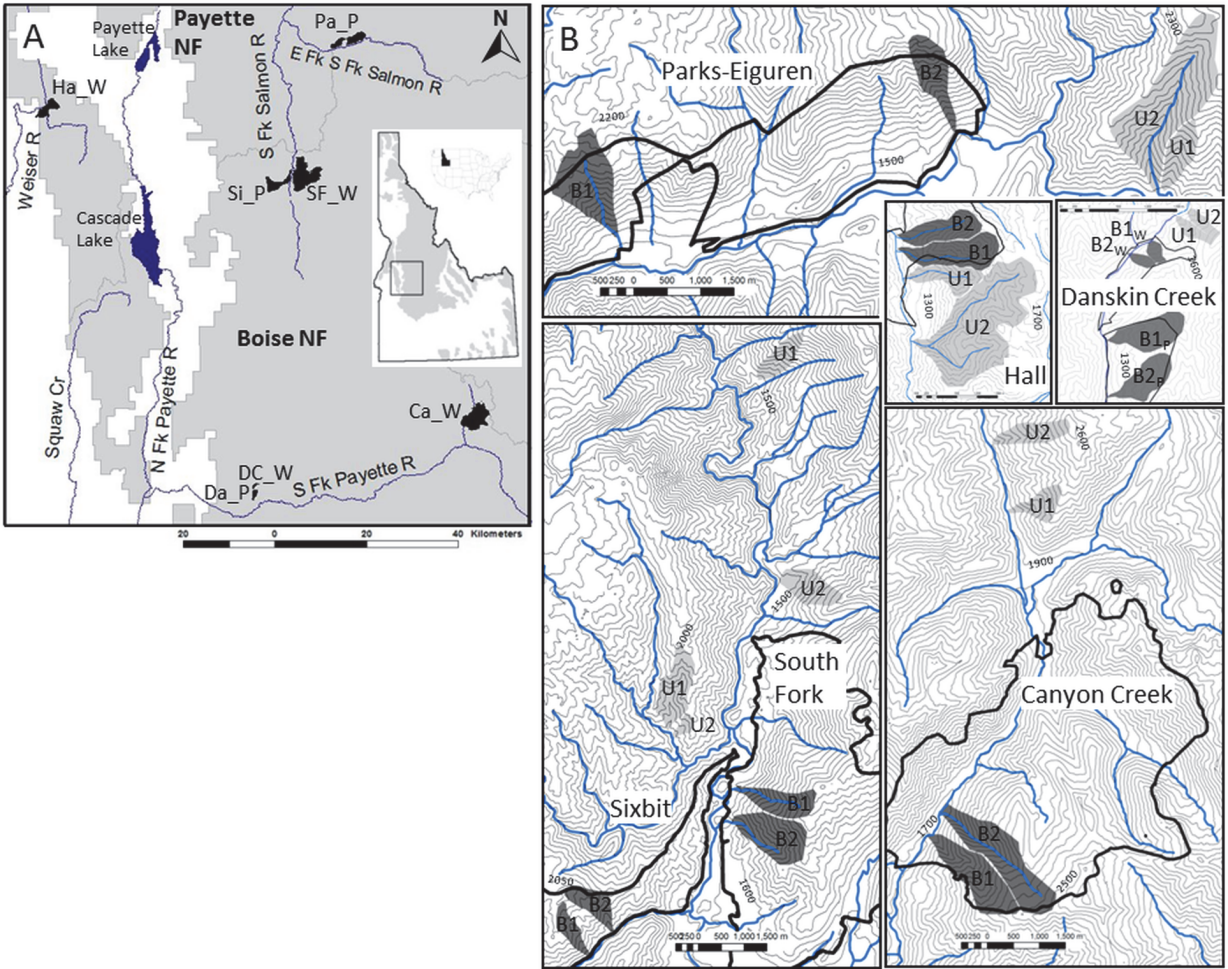
Study site and watershed locations. (A) Locations of prescribed burns (P) and wildfies (W) within the Boise and Payette National Forests (NF) of central Idaho. Da and DC—Danskin Creek, Pa—Parks-Eiguren, Si—Sixbit, Ca—Canyon Creek, Ha—Hall, SF—South Fork. (B) Locations of burned (B) and unburned (U) watersheds within each study site. At Danskin Creek, the 2002 wildfire and 2004 prescribed burn are in close proximity and share control watersheds. Elevation lines are at 50-m intervals; note the different scales between study sites. Thick black lines represent the fire perimeter. Blue lines represent streams; small first-order perennial or intermittent streams draining watersheds are not shown.

Our four wildfire sites (Hall, Canyon Creek, South Fork, Danskin Creek) and three spring prescribed burn sites (Danksin Creek, Sixbit, Parks-Eiguren) are located on the Boise and Payette National Forests in the Salmon River Mountains and West Mountains of central Idaho, USA (Fig 1A). Key characteristics of each site are summarized in Table 1. We conducted this field study with permissions from the USDA Forest Service ranger districts (RD) in which the study sites were located. Specifically, permissions were obtained from the Emmett RD office (Danskin Creek), the Lowman RD office (Canyon Creek), the Cascade RD office (South Fork, Sixbit), Krassel RD office (Parks-Eiguren), and the Council RD office (Hall). Our study did not involve any endangered or protected species. Study sites are located at 44°05’-44°57’N, 115°12’-116°21’W.

**Table 1:** Characteristics of wildfire and prescribed burn study sites.

Names of study sites	Canyon Creek	Hall	South Fork	Danskin Creek	Danskin Creek	Parks–Eiguren	Sixbit
Abbreviation	Ca	Ha	SF	DC	Da	Pa	Si
Fire type	Wildfire	Wildfire	Wildfire	Wildfire	Prescribed fire	Prescribed fire	Prescribed fire
Time of fire/burn	Aug 2003	Aug 2003	Aug–Oct 2003	July 2002	April 2004	May 2004	May 2004
Bedrock type	CRB	IBG	CRB	CRB	CRB	CRB	CRB
Mean max. annual/ max. Jan/ max. Jul temperature (°C)	11.3/ -2.8/ 25.9	16.6/ 0.4/ 32.7	12.8/ -1.2/ 28.1	16.8/ 1.1/ 32.8	see DC	12.8/ 0.8/ 26.7	see SF
Mean annual precipitation (cm) (% of total precip. from Nov through May)	33.5 (70)	60.9 (78)	81.9 (80)	62.5 (80)	see DC	68.2 (72)	see SF
Coordinates	115°14'W, 44°12'N	116°21'W, 44°50'N	115°44'W, 44°42'N	115°49'W, 44°5'N	115°49'W, 44°5'N	115°34'W, 44°58'N	115°44'W, 44°41'N
Elevation (m, B/U)	2100 / 2190	1420 / 1400	1940 / 1770	1440 / 1540	1410 / 1540	1880 / 1840	1960 / 1790
Aspect of watershed (B/U)	NW / W	W / W	W / SW	NW / NW	W-SW / NW	S-SE / S-SW	SE / S
Area of watershed (ha, B/U)	115 / 38	143 / 81	82 / 48	8 / 13	53 / 13	89 / 35	33 / 12
Distance between burned and unburned watersheds (m)	6800	600	9500	800	1600	8500	5500
Dominant conifer species (B/U)	PM, PP / AL, PM	PM / PM	PM / PM, PP	PM, PP / PM, PP	PM, PP / PM, PP	PM, PP / PM	PM, PP / PM
Overstory mortality^a^ (%, B/U)	84 / 5	12 / 1	48 / 1	36 / 16	5 / 0	7 / 2	3 / 0

Values of mean annual temperature and precipitation were obtained from the closest National Oceanic and Atmospheric Administration (NOAA) Cooperative measurement stations for each site in [29]. The stations are Stanley (Ca), Council (Ha), Garden Valley (DC/Da), Deadwood (SF and Si) and Yellowpine (Pa). Coordinates are for centers of burned watersheds (B1, see Fig 1B). B/U, comparison between burned (B1) and unburned (U1) watersheds (see Fig 1B); CRB, Columbia River basalt; IBG, Idaho batholith granitics; AL, *Abies lasiocarpa*; PM, *Pseudotsuga menziesii*; PP, *Pinus ponderosa*.

^a^ based on watershed pixels in a fire severity class higher than “low” (i.e., “low-moderate”, “moderate-high”, “high”) when using fire severity index dNBR (delta Normalized Burn Ratio). See Stephan et al. [6] for details.

Stream channels of studied watersheds are confined by relatively steep hill slopes (15–41°) and fringed by only a narrow strip (≤ 1 m width) of obligate riparian shrubs and herbs on each side. Birchleaf spiraea (*Spiraea betulifolia*) is a common and/or dominant understory species in the upland at all sites. Three of the wildfire sites had burned in the summer of 2003 and one site (Danskin Creek) had burned in 2002. The three spring burns occurred in April/ May of 2004 (Table 1).

In Stephan et al. [6], we assessed fire severity, defined as the aboveground and belowground consumption of organic matter [30], from Landsat satellite imagery before and one month (wildfires) to three months (spring burns) post-fire. As an index of fire severity, we used the delta Normalized Burn Ratio (dNBR) [31] mainly reflecting overstory mortality [32]. Delta NBR revealed significant and varying levels of overstory mortality in wildfire-burned watersheds, but little to no overstory mortality in the prescription-burned watersheds (Table 1). Based on ocular estimates on the ground, understory vegetation and forest floor was completely consumed over approximately 30 to 80% of the watersheds areas burned by the wildfires. In the prescription-burned watersheds, understory and forest floor was charred or consumed in relatively small patches (5 to 100 m^2^) covering less than one third of the total watershed area.

### Sample Collection and Analysis

Samples of soil, foliage of upland and obligate riparian plants, and in-stream moss were collected from a pair of burned/unburned watersheds (B1 and U1 in Fig 1B) from each of the three prescribed burn (P) and four wildfire (W) sites, respectively. At each site, samples were collected from several plots within the burned (B) watershed and the nearby unburned (U) watershed outside the fire perimeter. To ensure that paired watersheds within a given site had the highest likelihood of similar pre-fire N dynamics, watersheds chosen were similar in aspect, elevation (Table 1), and vegetation composition. Streamwater was collected from the mouths of the streams draining these watersheds and from one additional burned and unburned watershed per site (B2 and U2 in Fig 1B). Samples were collected in the two growing seasons (2004 and 2005) following the spring prescribed burns, three growing seasons (2004–2006) following the 2003 wildfires, and four growing seasons (2003–2006) after Danskin Creek wildfire. That is, sampling commenced 1–2 months after spring prescribed burns and 9 months after wildfires. All plant and moss samples were simultaneously analyzed for nitrogen concentrations [6] and nitrogen isotopic values. Due to fiscal and methodological limitations only a subset of soil and water samples collected for N concentration analysis could be analyzed for N isotopic values. Details of the sampling design are described in Stephan et al. [6]. Briefly, mineral soil (0–10 cm) and samples of live foliage from six upland understory species (four shrubs: *Spiraea betulifolia* Pall., *Physocarpus malvaceus* (Greene) Kuntze, *Symphoricarpos albus* (L.) Blake *and S*. *oreophilus A*. *Gray;* two sedges: *Carex geyeri* Boott and *C*. *concinnoides* Mack.) were collected from a total of four 10-m radius plots located within 25 m of either side of the stream per watershed. Live obligate riparian plant foliage of three shrub species (*Cornus stolonifera* Michx., *Rubus* sp., *Ribes* sp.) and two perennial forb species (*Circaea alpina* L., *Galium triflorum* Michx.) and terrestrial moss species growing on rocks in streams were collected from two plots per watershed, with each plot corresponding to a 20-m long stream reach. We composited similar plant species of the same genus (*Symphoricarpos* spp.; *Carex* spp.) or family (*Rubus/Ribes* spp.) when a single species did not occur on all sites. Within a single study site, however, the species was consistent between burned and unburned watersheds. All woody vascular plants sampled had resprouting capabilities. Per plot, each sample represents a respective composite of four to five soil cores, leaves of three to six individual plants, and three moss patches that were randomly chosen. Soil was collected in August 2004 and October 2005 for isotopic analysis of inorganic N. Plant foliage was collected in July/August to capture the cumulative effect by the end of the growing season. As an exception, in 2004, riparian foliage was collected in June. Moss was collected in May/June to capture the effect of N leached with spring runoff [6]. In July of 2005, fine roots (< 2 mm) of *S*. *betulifolia*, most consistently present in all plots per site relative to other species in this study, were collected from wildfire sites for N analyses. Streamwater was collected once in June 2006 for analysis of NO_3_^—^δ^15^N from one burned and one unburned watershed of each of the three 2003 wildfires. In general, the sampling period was limited to the growing season due to inaccessibility of the sites during winter and early spring.

### Plant δ^15^N

Plant material was freeze-dried except 2003 Danskin Creek foliage (dried at 70°C for 24 h). Dried material was ground to a fine powder with a ball mill, packed into tin capsules and analyzed for its δ^15^N value with continuous-flow direct combustion isotope ratio mass spectrometry (IRMS) following combustion in an elemental analyzer. Analyses of roots and foliage collected in 2004, 2005, and 2006 were carried out in the laboratory of Dr. R. Lee at the School of Biological Sciences at Washington State University (Isoprime [Micromass Ltd. Manchester, UK] with an EuroEA 3000 elemental analyzer [EuroVector S.p.A., Milan, Italy]). Foliage samples collected at Danskin Creek in 2003 and moss samples collected in 2004 and 2005 were analyzed in the University of Idaho Stable Isotope Laboratory (ISIL) (Finnigan Delta Plus [Finnigan MAT, Bremen, Germany] with a Carlo Erba NC 2500 elemental analyzer [CE Instruments, Milan, Italy]). Moss collected in 2006 was analyzed in the Laboratory for Biotechnology and Bioanalysis Stable Isotope Core at Washington State University (Delta PlusXP [Thermofinnigan, Bremen, Germany] with an ECS 4010 elemental analyzer [Costech Analytical, Valencia, California, USA]). In each laboratory, analytical precision for δ^15^N was ≤ 0.2‰ (standard deviation) between replicates of laboratory internal reference material and between replicates of actual sample material. Duplicate moss and foliage samples were run for inter-laboratory comparison at the laboratories involved in the respective analyses; standard deviations were ≤ 0.2‰ (n = 2) for moss samples and ≤ 0.1‰ (n = 8) for foliage samples. Thus, while not ideal, using multiple laboratories did not compromise data quality.

### Nitrate Reductase Activity

Foliar NRA was determined by the *in vivo* method [33] for *S*. *betulifolia* in each of the four established and two additional plots at wildfire and prescribed burn sites in June 2005 and at wildfire sites only in 2006. The method was adapted for use *in situ* (i.e., no vacuum, no DMSO, no boiling). Approximately 150–300 mg fresh leaf biomass was collected by cutting two 1-cm^2^ squares from each of a total of six young, fully developed leaves (from six individuals) per plot, and immediately incubated in assay medium in the dark at 25–30°C for ca. 60 minutes. Adding the color reagents stopped the reaction [34]. Spectrophotometric determination of the nitrite produced [34] was carried out upon returning to the laboratory within one week. Preliminary studies had shown that the color was stable for more than one week. Leaf material used in the assay was dried at 70°C for 24 h. NRA is expressed as μmol nitrite produced per hour and gram dry weight of tissue.

Despite diurnal changes of NRA [35], the NRA assays could not be performed at a constant time of day across all sites due to logistical reasons (all sites were remote). However, NRA in the burned and unburned watersheds for any given site was sampled within a 2 to 3 h time period to minimize the diurnal influence on NRA. In addition, at about half the sites, burned watersheds were sampled at times of lower light levels (i.e., earlier or later in the day and, thus, potentially lower NRA due to time of day) than unburned watersheds. This would remove potential bias that would have existed if burned watersheds had been consistently sampled later in the morning or earlier in the afternoon than their unburned counterparts.

### Soil and Streamwater δ^15^N

Fresh soil (sieved, 4-mm sieve) was extracted with 2 M KCl (Mallinckrodt Baker, Phillipsburg, NJ) while shaking for 1 h. Soil to extractant ratio was about 2:5 because soil inorganic N concentrations were very low. Soil extracts were filtered through Whatman No. 42 filters and extracts were stored frozen till analysis. A modified diffusion method by Stephan and Kavanagh [20] (based on Holmes et al. [36]) was used to isolate and concentrate NH_4_^+^-N on filter discs that were analyzed for δ^15^N at ISIL. Soil extracts from August 2004 contained ca. 40 μg NH_4_^+^-N in volumes of 20 to 50 mL, and extracts from October 2005 contained on average 48 μg (range 10–110 μg) NH_4_^+^-N in ca. 100 ml extract. Samples were diffused during a 6-d diffusion period at room temperature or at 34°C, respectively. Recoveries of sample-NH_4_^+^-N, calculated as: [recovered N] / ([expected target N] + [contaminant N]) were on average 96% ± 0.2 (SD) and 90% ± 10 for August 2004 and October 2005 diffusions, respectively. Samples that had recoveries of < 99% of expected N were corrected for fractionation during incomplete recovery [20,36]. Contaminant-NH_4_^+^-N contributed from reagents was quantified as described by Stephan and Kavanagh [20] and found negligible. Therefore, no correction to obtain the true target-NH_4_^+^-δ^15^N was necessary.

Analysis of soil extract NO_3_^—^δ^15^N was not possible in August of 2004 because the extracts contained too little NO_3_^—^N for accurate analysis with the IRMS. In October 2005, a larger soil volume was extracted (75 g fresh soil in 150 ml 2 M KCL). Still, only half of the samples contained sufficient NO_3_^—^N to be diffused (average 47 μg, range 13–150 μg). Diffusions for NO_3_^—^N were carried out for 6 d at room temperature after NH_4_^+^-N had been trapped (see above). The method is modified from Sigman et al. [37] and is described in Stephan & Kavanagh [20]. During sequential diffusions, NH_4_^+^-N that is not trapped in the first step will be enriched and might be carried over into the subsequent nitrate diffusion. We assumed this had happened with NH_4_^+^-N that was not recovered in the first diffusion step. Additionally, reagents have been shown to contribute significant amounts of contaminant-NO_3_^—^N [11]. Sample recovery (including assumed carry-over of NH_4_^+^-N and reagent-contaminant-NO_3_^—^N) was 75% ± 13 (SD). We corrected potential error due to the amounts and isotopic values of carry-over NH_4_^+^-N and reagent-NO_3_^—^N, and fractionation due to incomplete sample-NO_3_^—^N recovery, and calculated the true target NO_3_^—^δ^15^N as detailed in Stephan & Kavanagh [20].

Streamwater from the three 2003 wildfire sites was analyzed for NO_3_^—^δ^15^N using the denitrifier method [37] at the Woods Hole Oceanographic Institute, Woods Hole, Massachusetts, USA. For each of the six water samples, analysis was conducted on three subsamples to ensure analytical precision; the SD between the three subsamples was < 0.18‰. More extensive analysis of streamwater NO_3_^—^δ^15^N was not possible due to the high cost associated with analysis by service laboratories using the denitrifier method.

All sample materials were stored on ice in a cooler during the collection period and transport to the laboratory. Due to the remoteness of the field sites all sample processing in the laboratory commenced one to five days after field collection.

### Statistical Analysis

The study design is comparable to a block design (site = block, watershed = plot). Prior to statistical analysis, values of soil and vegetation, and in-stream moss samples taken at the four or two plots (i.e., statistical subplots) within watersheds were averaged. Data were transformed if necessary and subjected to analyses of variance (ANOVA) with linear mixed-effect models in SAS (SAS 9.1, SAS Institute Inc., Cary, NC, USA). The objectives of the statistical analysis were to test for the absence of a) a difference between burned and unburned watersheds for wildfires and spring prescribed burns, respectively, and b) a difference in the magnitude of the post-fire response between wildfires and prescribed burns. We also analyzed the effect of time (sampling date) on ^15^N values but exercised care when interpreting these data due to the low temporal resolution of data, inter-laboratory variability (though small), and/or inconsistencies in sample processing between different years. We assessed fire effects based on the assumption that a burned watershed and the corresponding unburned watershed had been similar pre-fire. This assumption was reasonable due to our careful pairing of burned/unburned watersheds in each site so that watershed pairs were similar in biotic and abiotic characteristics including aspect, elevation, and dominant vegetation in overstory as well as understory (Table 1).

In our mixed-effect models, sites were specified as random effects. Consequently, inference drawn from this study is not limited to the very sites studied, but applies to similar wildfires and spring prescribed burns in mid-elevation headwater watersheds within this ecoregion. In addition to site, the watersheds nested within each site were included as random effect if permitted by the data structure. This allowed for random interactions between site and treatment, i.e., the magnitude of the burn effect could vary between sites and/or the two watersheds within each site could differ from each other due to, e.g., slight variation in elevation, slope or (pre-fire) soil characteristics. Serial correlation between δ^15^N values of samples collected through time was assumed and accounted for with repeated measures. In summary, only those treatment effects that were sufficiently strong in all sites were detected.

Regressions were carried out using general linear models. Here we used plot values rather than watershed averages because the within-watershed variation was larger than the variation between watersheds. This resulted in lower coefficients of determination (R^2^) and larger P-values than when watershed values were used.

Data from the Danskin Creek wildfire, that had burned one year prior to the other three wildfire sites, was analyzed in the appropriate post-fire season in the analysis of NRA, foliar δ^15^N, and root δ^15^N. Due to the high short-term temporal variability in soil, Danskin Creek wildfire data on soil δ^15^N was analyzed together with data from the other wildfires collected at the same sample date.

In the results, N isotope data are generally presented in the order of 1) burned vs. unburned in prescribed burn sites (PB vs. PU), 2) burned vs. unburned in wildfire sites (WB vs. WU), and 3) prescription-burned watersheds vs. wildfire-burned watersheds (PB vs. WB) for each ecosystem N pool. Data are presented per sampling date or as the average of a given growing season post fire (also referred to as post-fire year). Model P-values and/or P-values of pairwise comparisons (burned vs. unburned, prescribed burn vs. wildfire) for given sampling dates are presented. Means, standard deviations, and standard errors presented in the text, graphs, and tables are based on untransformed raw data.

## Results

### Plant δ^15^N

**Fig 2:**
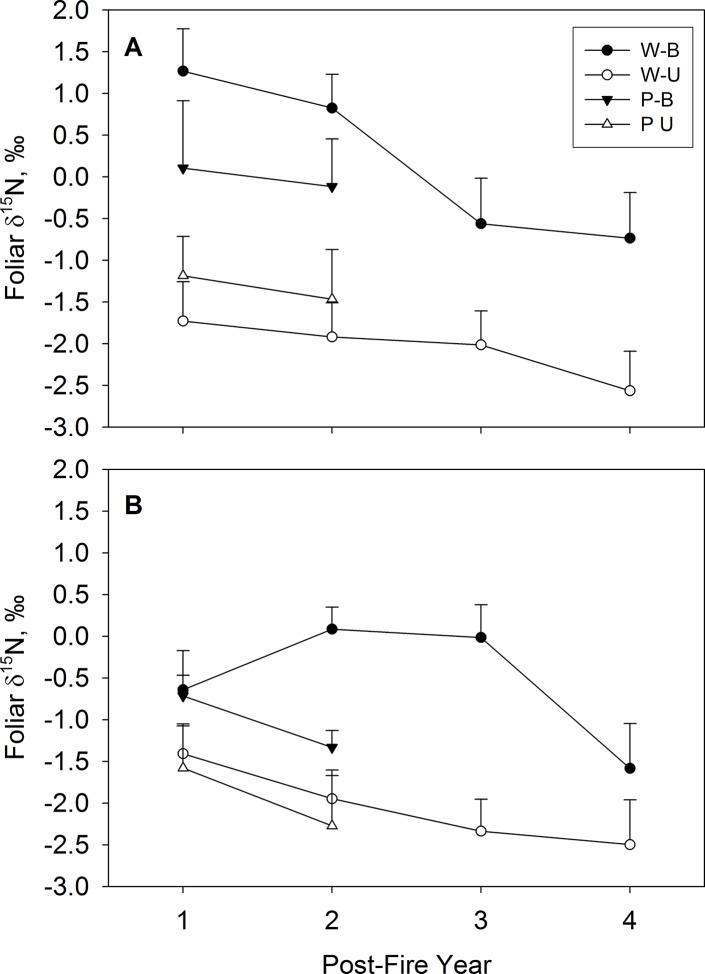
Foliar δ^15^N values in burned (B) and unburned (U) watersheds of prescribed burn (P) and wildfire (W) sites. Foliar δ^15^N values are averaged across all (A) upland species and (B) riparian species. Error bars represent 1 SE across four species. Each species’ value was obtained by first averaging across sites; the variability across sites per species is presented in Table 2. Data for the fourth post-fire year represents only the Danskin Creek wildfire site. Note that averaging across species obscures species × treatment interactions to some extent (see text).

Following the fire, foliar δ^15^N of upland plants was generally higher in burned than unburned plots, but the magnitude of enrichment differed between wildfire and prescription-burned sites (Table 2). All upland species from WB plots had significantly higher foliar δ^15^N than in WU plots across all three post-fire years (P = 0.01). All species showed a consistent pattern in foliar δ^15^N with no statistically significant interaction effects among species, treatment, and post-fire year. The difference in foliar δ^15^N between WB and WU persisted for several years. The average differences were 3.0 ‰ and 2.8 ‰ in the first and second post-fire year, respectively. These differences decreased to 1.4 ‰ by post-fire year 3 (P < 0.001). In addition, data from the Danskin Creek wildfire site indicated that the burned-unburned differences in post-fire year 3 persisted in post-fire year 4 (Fig 2A).

**Table 2:** Foliar δ^15^N (‰) of all species and root δ^15^N (‰) and nitrate reductase activity (NRA, μmol g^-1^ h^-1^) of *S*. *betulifolia* in burned and unburned watersheds in each post-fire year.

	Wildfires						Prescribed Burns			
	Post-fire year 1		Post-fire year 2		Post-fire year 3		Post-fire year 1		Post-fire year 2	
	Burned	Unburned	Burned	Unburned	Burned	Unburned	Burned	Unburned	Burned	Unburned
Upland										
*Carex spp*.^x^	1.3 (1.5)* ^a^	-3.9 (0.3)	-0.2 (0.1) * ^a^ ^b^	-3.1 (0.6)	-1.7 (0.5) ^b^	-3.1 (0.5)	-2.3 (0.4) ^a^	-2.5 (0.4)	-1.8 (0.3) ° ^a^	-2.9 (0.3)
*Physocarpus malvaceus*	-0.2 (0.6) ^a^ ^b^	-1.6 (0.9)	1.0 (0.5) * ^a^	-1.2 (0.4)	-1.3 (0.6) ^b^	-2.0 (0.6)	1.1 (0.3) * ^a^	-0.5 (0.01)	0.6 (0.4) ° ^a^	-0.8 (0.6)
*Symphoricarpos spp*.^y^	2.2 (0.8) * ^a^	-1.7 (0.8)	0.9 (0.1) * ^a^ ^b^	-1.9 (0.8)	0.3 (0.6) ° ^b^	-1.7 (1.3)	0.5 (0.4) * ^a^	-1.2 (0.5)	0.0 (0.2) * ^a^	-2.0 (0.9)
*Spiraea betulifolia*	1.7 (0.6) * ^a^	-0.7 (0.5)	1.7 (0.2) * ^a^	-1.5 (0.3)	0.4 (0.4) * ^b^	-1.2 (0.4)	1.1 (0.5) ° ^a^	-0.5 (0.6)	0.7 (0.6) ^a^	-0.2 (0.7)
*S*. *betulifolia* root^z^	nc	nc	-1.0 (0.1) ^a^	-2.7 (0.5)	-2.3	-2.4	nc	nc	nc	nc
*S*. *betulifolia* NRA^z^	nc	nc	1.6 (0.3) ^a^	0.5 (0.1) ^b^	0.6 (0.1)	0.5 (0.1)	nc	nc	0.8 (0.3)	0.7 (0.2)
Riparian										
*Galium triflorum*	0.5 (0.6) * ^a^	-1.4 (0.4)	0.3 (0.5) * ^a^	-2.0 (0.3)	-0.8 (0.6) * ^b^	-2.6 (0.4)	0.0 (0.3) * ^a^	-1.6 (0.5)	-1.4 (0.2) ° ^b^	-2.4 (0.4)
*Circaea alpina*	-0.6 (0.9) ^a^	-2.0 (0.7)	0.4 (0.8) * ^a^	-2.7 (0.5)	1.0 (1.5) * ^a^	-3.3 (0.5)	-0.7 (0.4) * ^a^	-2.7 (0.2)	-1.8 (0.4) * ^a^	-3.8 (0.6)
*Cornus stolonifera*	-1.8 (0.4) ^a^	-1.8 (0.5)	-0.7 (0.5) ^b^	-1.9 (0.5)	-0.4 (0.5) ° ^b^	-1.8 (0.6)	-1.2 (0.6) ^a^	-1.8 (0.8)	-1.4 (0.3) ^a^	-2.4 (0.6)
*Rubus/Ribes spp*.	-0.8 (0.4) ^a^	-0.5 (0.4)	0.3 (0.5) ° ^a^	-1.3 (0.7)	0.1 (0.7) * ^a^	-1.7 (0.5)	-0.9 (0.5) ^a^	-0.2 (0.9)	-0.8 (0.5) ^a^	-0.5 (0.5)
In-stream moss	0.2 (0.2)^a^	-0.3 (0.5)	1.8 (0.4) * ^b^	0.5 (0.4)	0.9 (0.2) * ^a^ ^b^	-1.2 (0.2)	0.0 (0.7) ^a^	1.3 (0.7)	0.9 (0.5) ^a^	0.0 (0.5)

One SE is given in parentheses (n = 3 or 4 sites, each n represents the average of four (upland foliage) or two (riparian foliage, moss) subsamples).

*/° P ≤ 0.05/0.10 for individual pairwise comparisons of treatment for a given species and post-fire year.

^a,b^ P < 0.05 for individual pairwise comparison between post-fire years of burned watersheds for each species and for wildfire and spring prescribed burns, separately. Values from unburned watersheds did not differ between years.

^x^ *C*. *geyeri* and *C*. *concinnoides*;

^y^ *S*. *albus* and *S*. *oreophilus*

^z^ Data in wildfire sites of post-fire year 2 comprise the three 2003 wildfire sites, in post-fire year 3 just the Danskin Creek site.

nc Data not collected.

Nitrogen in *S*. *betulifolia* roots was significantly enriched in ^15^N by 1.7 ‰ (P = 0.049) in burned relative to unburned plots of the three 2003 wildfire sites in 2005 (post-fire year 2). However, there was no difference in root-δ^15^N between the burned and unburned plots at the Danskin Creek wildfire site in 2005 (post-fire year 3) (Table 2). Roots were isotopically depleted relative to *S*. *betulifolia* foliage in WU plots (absolute difference in δ^15^N 1.2 ‰, P = 0.01) and more so in WB plots (absolute difference in δ^15^N 2.7 ‰, P < 0.001). *S*. *betulifolia* root N concentrations, averaged across the four wildfire sites, were 1.1% and 0.95% at burned and unburned plots, respectively (P = 0.39).

In prescribed burn sites there was a significant burn effect on foliar δ^15^N values for all species sampled (P = 0.04), with the exception of *Carex* spp. in post-fire year 1 (Table 2). The difference in average isotopic values in PB vs. PU plots was 1.3 ‰ in both post-fire year 1 and post-fire year 2 and, thus, significantly smaller (P = 0.01) than the respective differences after wildfire (Fig 2A). In all unburned plots (prescribed burn and wildfire sites), δ^15^N values of species did not differ between different post-fire years (Fig 2A). Foliar N isotopic values of individual species did not differ between WU and PU with a possible exception of *S*. *betulifolia* in post-fire year 2 (P = 0.05, Table 2).

The foliar δ^15^N of riparian species responded differently than that of upland species (Fig 2A and 2B); specifically, there was a delay or absence of foliar ^15^N enrichment in riparian shrubs. In wildfire sites, there was a significant treatment effect (P = 0.007), treatment × post-fire year interaction (P = 0.005) and treatment × species interaction (P = 0.002). Foliar δ^15^N values were not consistently higher across all riparian species in WB relative to WU in post-fire year 1 (P = 0.16); only herbaceous *G*. *triflorum* was significantly enriched in ^15^N WB relative to WU (Table 2). However, in post-fire year 2 and post-fire year 3, higher δ^15^N values in WB relative to WU were significant across all species, with absolute ^15^N enrichment averaged across all species of 2.1 ‰ (P = 0.002) and 2.4 ‰, (P = 0.001), respectively. Data for post-fire year 4, available for Danskin Creek wildfire only, showed a 1 ‰ enrichment in δ^15^N of WB relative to WU (Fig 2B).

In prescribed burn sites, there was a significant treatment × species interaction (P = 0.003). Riparian forbs had higher post-fire δ^15^N values in PB relative to PU plots for both post-fire years studied but there was no treatment effect in riparian shrubs (Table 2).

When comparing riparian foliage δ^15^N values after wildfires and spring prescribed burns, there was no difference between PB and WB in post-fire year 1. However, in post-fire year 2, riparian foliage in WB had significantly higher δ^15^N values than in PB (absolute difference across all species 1.4 ‰, P < 0.001). There was no difference in isotopic values between WU and PU at any sampling date. Interestingly, foliar isotopic values of riparian plants did not differ from those of upland plants in unburned plots despite differing species compositions.

Delta^15^N values of in-stream moss in wildfire sites responded in a similar way as riparian plant foliage in these sites. Delta^15^N values of moss in WB were not statistically significantly different from WU in post-fire year 1 but in post-fire year 2 and post-fire year 3 a treatment effect was detected (Table 2). Data from Danskin Creek wildfire (not shown) indicated that the treatment effect persisted in the fourth post-fire year. Moss in PB plots had δ^15^N values that were not different from PU plots at any sampling date (Table 2).

### Nitrate Reductase Activity

**Fig 3:**
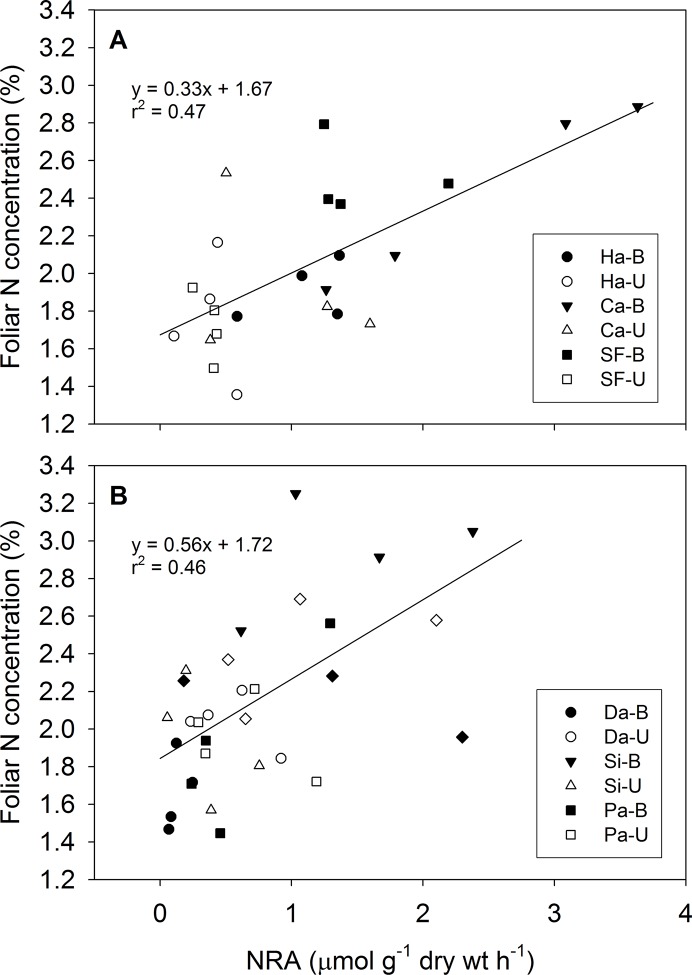
Relationship between foliar nitrate reductase activity (NRA) and foliar N concentration of *S*. *betulifolia*. Relationships are shown for (A) the three 2003 wildfire sites and (B) spring prescribed burn sites in the second post-fire year. Open and filled symbols represent unburned (U) and burned (B) plots, respectively. Site abbreviations: Da and DC—Danskin Creek, Pa—Parks-Eiguren, Si—Sixbit, Ca—Canyon Creek, Ha—Hall, SF—South Fork.

NRA varied with treatment, fire type, and post-fire year. Foliage of *S*. *betulifolia* collected in June of post-fire year 2 had higher foliar NRA in burned plots relative to unburned plots across the three 2003 wildfire sites (P = 0.04) but not across all three prescribed burn sites (P = 0.78). In post-fire year 3, there were no differences in NRA between WB and WU (P = 0.50) (Table 2), and this was also the case at the Danskin Creek wildfire site in post-fire year 4 (data not shown). Foliar NRA and N concentration of *S*. *betulifolia* were positively correlated in prescribed burn sites (R^2^ = 0.46, P < 0.001) and wildfire sites (R^2^ = 0.47, P < 0.001) (Fig 3A and 3B).

### Soil and Streamwater δ^15^N

**Fig 4:**
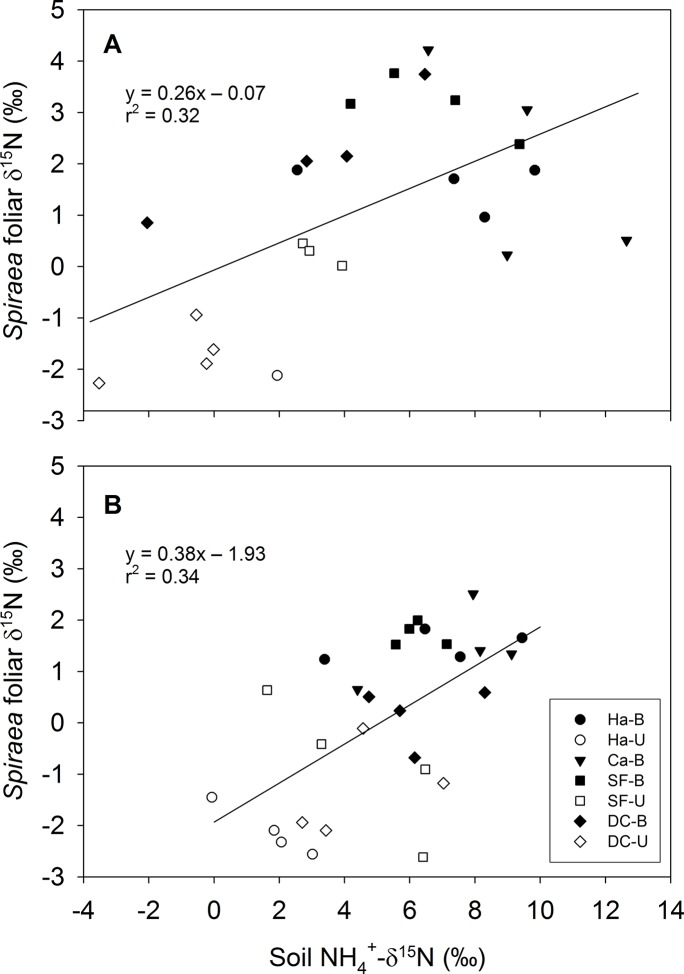
Relationship between soil NH_4_^+^-δ^15^N and foliar δ^15^N of *S*. *betulifolia* in the wildfire sites. Relationships are shown for soil NH_4_^+^-δ^15^N collected in (A) August 2004 and (B) October 2005. Each data point represents one plot since variability within watersheds was higher than variation between watersheds. Open and filled symbols represent unburned (U) and burned (B) plots, respectively. Site abbreviations: DC—Danskin Creek, Ca—Canyon Creek, Ha—Hall, SF—South Fork. No data was available for Ca-U.

Soil inorganic N was significantly enriched in ^15^N in WB relative to WU but no treatment effect was found in prescribed burn sites (Table 3). Across all four wildfire sites, ^15^N of NH_4_^+^ in soils extracts from August 2004 (post-fire year 1 for three sites, post-fire year 2 for Danskin Creek) was almost 6 ‰ higher in WB relative to WU (P = 0.02), but there was no consistent pattern across prescribed burn sites (P = 0.24, Table 3). This pattern was also found in October 2005 except that in wildfire sites the absolute difference between WB and WU was smaller (P = 0.07), namely 3.2 ‰ ± 0.8 (SE), compared to 5.8 ‰ ± 1.2 in 2004 (Table 3). In both 2004 and 2005, foliar δ^15^N of *S*. *betulifolia* correlated positively (R^2^ = 0.32, P = 0.004; R^2^ = 0.34, P = 0.001, respectively) with soil δ^15^NH_4_^+^-N (Fig 4A and 4B) (since the foliage of the other upland species had foliar δ^15^N pattern similar to *S*. *betulifolia*, only *S*. *betulifolia* is presented). Soil NO_3_^-^ data (Table 3) available only for October 2005 had many missing data points due to low N content in extracts from unburned soil preventing the use of the diffusion technique. Statistical analysis of fire treatment effects was therefore not possible; however, it could be shown that soil NO_3_^-^ was isotopically depleted relative to soil NH_4_^+^ by on average 4.5 ‰ (P = 0.002).

**Table 3:** Mean δ^15^N (‰) values of soil inorganic N in burned and unburned watersheds at two sample dates.

	Wildfires		Prescribed burns	
	Burned	Unburned	Burned	Unburned
NH_4_^+^ δ^15^N, Aug 2004	6.5 (1.4)^a^	0.7 (0.7) ^b^	2.5 (0.4)	1.2 (0.9)
NH_4_^+^ δ^15^N, Oct 2005	7.0 (0.2) ^a^	3.8 (0.6)^b^	6.4 (1.4)	5.2 (0.6)
NO_3_^-^ δ^15^N, Oct 2005	2.4 (1.4)	-0.2^x^	1.9 (1.1)	0.4 (3.1)^y^

One SE is given in parentheses (n = 4 sites), each n represents the average of generally four plots (subsamples).

^x^ Value represents only one plot from one site

^y^ Value represents only six plots from two sites, hence statistical analysis of treatment differences not performed

^a,b^ significant treatment difference at P ≤ 0.05.

Streamwater NO_3_^—^δ^15^N of the burned watershed was higher than that of the corresponding unburned watershed at each of the three 2003 wildfire sites. Values for streamwater NO_3_^—^δ^15^N at the burned and unburned watershed of Hall, Canyon Creek and South Fork sites, respectively, were 4.0‰ vs. -10.7‰; 3.6‰ vs. 2.8‰, and 3.4‰ vs. 2.6‰. Thus, streamwater NO_3_^—^δ^15^N was consistently higher from burned watersheds than unburned watersheds. Given the small sample size of n = 3, statistical analysis has limited power. However, burned-unburned differences were statistically significant across sites if the value from the unburned watershed at the Hall site was removed as statistical outlier.

## Discussion

This study indicates the tight coupling of terrestrial and aquatic ecosystem biogeochemistry after forest fire events and the importance of fire severity on N cycling processes. This is supported by ^15^N enrichment in both terrestrial (soil NH_4_^+^ and upland and riparian plant foliage) and aquatic (streamwater NO_3_^-^ and moss) N pools after wildfires whereas low-severity prescribed burn effects were limited to terrestrial N pools. These findings are supported by companion studies, investigating soil microbial N transformation processes [5] and N concentrations in soil, foliage, streamwater, and in-stream moss [6] at the same study sites. An increase in ecosystem N availability in burned relative to unburned watersheds was initiated by reduced microbial uptake in the soil [5,11] and concurred with increased plant N concentrations and NO_3_^-^ leaching into highly oligotrophic headwater streams [6]. Using the N isotope signal, it is evident that the increased foliar N concentrations of upland plants and in-stream moss in burned watersheds were the result of high availability of inorganic N in soil and high NO_3_^-^ availability in streamwater [6].

This research demonstrates that ^15^N values of ecosystem components at natural abundance are a useful indicator of post-fire N biogeochemistry and the magnitude of alteration of N cycling processes. The most parsimonious explanation for the elevated plant foliage δ^15^N and in-stream moss δ^15^N is that they reflect the δ^15^N of their N source. Grogan et al. [24] reached a similar conclusion following measurements of foliar isotopic enrichment after wildfire in a Californian bishop pine forest. Source N isotopic enrichment was further supported by our direct measurements of soil δ^15^NH_4_^—^N, and possibly, δ^15^NO_3_^—^N of streamwater, although a larger sample size would be needed to conclusively support the latter.

### Fire Effects on Terrestrial N cycling

Causes of isotopic enrichment of soil inorganic N are well established in the literature. Briefly, the most likely mechanism for soil inorganic ^15^N enrichment post-fire is fractionation occurring during volatilization and/or combustion of N in organic matter leading to preferential loss of ^14^N [2,38,39]. This has been experimentally demonstrated by heating organic and mineral soil in a muffle furnace at different temperatures [28] where the highest isotopic enrichment of residual N (by 2.5 ‰) occurred at the highest combustion temperatures (400°C) and durations (60 min). Furthermore, enrichment correlated with proportion of N lost. These experimental results were corroborated by preliminary studies [40] in one of our wildfire study sites (Danskin Creek), where N in ash was enriched by about 4 ‰ relative to the organic matter of the unburned forest floor.

Enriched foliar δ^15^N has also been linked to increased rates of net nitrification [41] with the theoretical foundation provided in a model by Shearer et al. [42]. Due to the larger fractionation associated with nitrification than with microbial NH_4_^+^ immobilization, the residual NH_4_^+^ pool becomes enriched with an increasing proportion of NH_4_^+^ nitrified rather than immobilized. As source NH_4_^+^ becomes enriched, NO_3_^-^ produced from it by nitrification (while being depleted relative to source NH_4_^+^) will also become enriched with an increasing proportion of NH_4_^+^ nitrified rather than immobilized [42]. Furthermore, if NO_3_^-^ is lost via leaching, the residual N will be enriched relative to the NO_3_^-^ lost, causing additional increases in δ^15^N of shallow-rooted plants. Both increased net nitrification and leaching losses of NO_3_^-^ are generally observed in burned areas or after other vegetation disturbances, and increased foliar δ^15^N has been attributed to these mechanisms [21,22,24].

Any combination of the above mechanisms could explain our observed ^15^N enrichment of inorganic soil and subsequently contribute to foliar ^15^N enrichment of upland and riparian plants. Simultaneously, these mechanisms can explain differences in foliar isotopic response between wildfires and spring prescribed burns since they are either directly linked to fire severity (proportion of N volatilized or combusted) or altered in relation to fire severity (e.g. net nitrification).

Lower combustion temperatures [38] and incomplete removal of the forest floor [43] are commonly observed with spring prescribed burns. We did not measure the former but did observe the latter. Thus, with our spring prescribed burns it is likely that less volatilization of ^14^N had occurred and resprouting plants could still acquire N mineralized from the partially charred, residual organic horizon. This is supported by small changes in gross ammonification rates and microbial NH_4_^+^ uptake after spring burns relative to controls and relative to wildfire sites (in which ammonification and NH_4_^+^ uptake were reduced relative to controls) measured in October of post-fire year 2 [5]. However, after both wildfires and spring prescribed burns, we did observe higher net nitrification on burned sites than in unburned sites in October of post-fire year 2, although not due to increased gross nitrification but due to reduced gross NO_3_^-^ immobilization [5]. These results are consistent with soil inorganic N concentrations measured at that time [6]. While we did not measure gross N transformation rates at other times, soil NH_4_^+^ and NO_3_^-^ concentrations were significantly increased in PB relative to PU and WB relative to WU, especially in post-fire year 1 [6]. At that time, soil NH_4_^+^-N concentrations were increased about four to six-fold in both PB and WB relative to unburned plots. While there was no statistically significant difference between PB and WB, there was a trend for lower NH_4_^+^-concentrations in PB relative to WB. Soil NO_3_^—^N concentrations were mostly below detection limit in PU and WU, but could be detected in PB and WB. Soil NO_3_^—^N concentrations were statistically significantly lower in PB relative to WB. The differing magnitudes of soil inorganic N concentration increases in WB and PB were likely driven by burn severity rather than burning season. This conclusion was based on a) our unpublished data of a severe spring prescribed burn that produced N concentration increases in soil similar to those after wildfires and b) a positive correlation between wildfire-burned watershed area (as a measure of severity) and streamwater NO_3_^-^ concentrations (Fig 4 in [6]). In addition, Turner et al. [44] found that vegetation cover following revegetation after fire influences soil N (measured as *in situ* annual net N mineralization using resin cores). At fine spatial scales, soil ammonium and nitrate concentrations were negatively correlated with live vegetation cover and unburned litter cover (i.e. inorganic N sinks); a positive correlation was found with the presence of bare mineral soil. While we did not measure vegetation recovery, it is reasonable to assume that revegetation occurs more readily after low severity spring prescribed burning that after wildfire [45].

The hypothesis that soil inorganic N enrichment is due to leaching of isotopically lighter NO_3_^-^ is supported by the very high streamwater NO_3_^-^ concentrations that were found in wildfire-burned watersheds relative to unburned watersheds [6]. In spring samples of all three post-fire years, NO_3_^-^ concentrations in WB were at least one order of magnitude higher than in WU. Leaching of NO_3_^-^ into streams occurred during the three post-fire seasons at about equal magnitude in WB, whereas little or no leaching occurred after spring prescribed burns as indicated by similar streamwater NO_3_^-^ concentrations in PB and PU [6].

Since the patterns of streamwater NO_3_^-^ concentrations post-fire (i.e., the duration and magnitude of concentration increases) corresponded with the pattern in foliar δ^15^N, leaching could in part explain the differences in foliar isotopic response between wildfires and spring prescribed burns. The most decisive evidence of post-fire enriched plant foliage being caused by uptake of enriched source N is the presence of post-fire ^15^N enriched plant available NH_4_^+^-N (Table 3) and the significant positive correlation between foliar δ^15^N of *S*. *betulifolia* and δ^15^N of soil NH_4_^+^-N (Fig 4). Unfortunately, no conclusive isotopic data for soil NO_3_^-^ was available. However, if soil NH_4_^+^ is enriched post-fire, NO_3_^-^ derived from it should also to be enriched relative to unburned conditions.

Plant uptake of abundant soil inorganic N was also supported by higher foliar N concentrations [6] and higher NRA in burned relative to unburned watersheds (Table 2). Upland foliage N concentrations were significantly higher in both PB and WB relative to PU and WU by 44% and 51%, respectively, in post-fire year 1. However, despite stronger isotopic enrichment in WB than PB, plant foliage N concentrations were not significantly different between PB and WB and no treatment effect on N concentrations was detected in later post-fire years in either wildfire or prescribed burn sites [6] despite persistent ^15^N enrichment. Nitrate reductase activity, and thus NO_3_^-^ use by plants, was significantly higher after wildfire relative to prescribed burns and unburned controls (Table 2) and correlated moderately well with foliar N concentration in prescribed burn sites and wildfire sites (Fig. 3A and 3B), thus indicating assimilation of abundantly available NO_3_^-^ -N after wildfire. Stewart et al. [46] also found higher NRA after recent (0.3 to 2.5 years) fire relative to sites burned 4 to 23 years previously.

Overall, the relatively low coefficients of determination in regressions of foliar δ^15^N against soil NH_4_^+^- δ^15^N (Fig 4) for wildfire sites underscore that the isotopic values of source N are not the only determinants of foliar-δ^15^N. Several other factors can influence plant foliar δ^15^N [17]: origin(s) of source N (soil N, precipitation, foliar N uptake, N_2_-fixation), rooting depth, influences of mycorrhizal symbioses, and fractionations during N uptake by plants. While we could discount N from precipitation, pollution, and N_2_-fixation, the other factors might have influenced foliar δ^15^N. In our study region, N in precipitation is very low (1.4 kg N ha^-1^ y^-1^) [47] and about one to two orders of magnitude lower than the annual requirement by understory vegetation [48]. Foliar uptake of NO_x_ and NH_3_, which can be substantial in areas with air pollution [49], is likely negligible due to the high air quality in our remote study area. N-fixing plants were also rare so that N derived from this source would be a minor proportion of soil N. None of these potential N sources differed between burned and unburned watersheds so that our assumption of soil N (derived from mineralization) as the dominant N source for plants is reasonable.

Foliar δ^15^N in burned watersheds became more similar to that of bulk mineral soil (0–10 cm) (ranging from 2.6 to 4.9 ‰; [40]). This would be consistent with deeper root location after fire [50]. In addition, high post-fire soil inorganic N availability [6] and therefore potentially reduced degrees of mycorrhizal infection [51] could have contributed to increased foliar isotopic values [52,53] in burned relative to unburned areas. Fractionation during N uptake might have occurred in some wildfire-burned locations where plants might not have been N-limited. However, with fractionation during N uptake, values of foliar δ^15^N would have been expected to decrease relative to N-limited conditions of unburned areas. Since we found the opposite, fractionation during uptake would only have decreased the magnitude of post-fire foliar enrichment that would have been otherwise observed.

In summary, the observed pattern in foliar isotopic values could be the result of several superimposed processes [23,54]. However, we maintain that fire-induced enrichment of source inorganic N via volatilization and NO_3_^-^ leaching, and subsequent cycling of enriched residual N are likely the major factors in the foliar isotopic enrichment after fire.

### Fire Effects on Aquatic and Riparian N Cycling

Saito et al. [28] suggested that the fire-induced changes in bulk soil δ^15^N may be a useful means for tracing impacts of fire on the aquatic food web. To our knowledge, our study is the first to show that the fire-induced change in soil isotopic signature can be traced to streamwater and in-stream moss and, thus, to demonstrate close terrestrial-aquatic linkages. Spencer et al. [7] found higher δ^15^N values in fish and aquatic macroinvertebrates after a large wildfire in Montana, and attributed this to a change from terrestrial to aquatic food sources rather than a change in terrestrial food’s δ^15^N. It is possible that a terrestrial fire signal contributed to the aquatic of ^15^N values reported by Spencer et al. [7]. Silins et al. [55] also reported higher δ^15^N of aquatic macroinvertebrates in burned watersheds relative to those in unburned reference watersheds in Alberta, Canada. In our study, riparian plants in WB (relative to WU) had higher foliar δ^15^N values, particularly in post-fire year 2 and 3 but foliar N concentrations in WB were similar to WU (and concentrations in PB were similar to PU in all post-fire years) [6]. This uptake of post-fire available N highlights the function of riparian plants as buffers between upland and aquatic processes that, by studying foliar N concentrations alone, might have been overlooked. The in-stream moss of the wildfire sites had higher foliar N concentrations in burned vs unburned watersheds starting with post-fire year 1 [6]. Moss N concentrations were 15% higher (P = 0.05) in WB relative to WU on all sampling dates but did not differ between PB and PU on any sampling date [6]. Despite the higher moss N concentrations in post-fire year 1 (WB relative to WU), moss did not show higher δ^15^N until post-fire year 2. In ^15^N tracer additions to streams, moss was identified as an important sink for both NH_4_^+^ and NO_3_^-^ [56]. In our WB watersheds, streamwater NO_3_^-^ but not NH_4_^+^ concentrations were higher relative to WU [6], suggesting that moss foliar δ^15^N enrichment resulted from the uptake of abundant and isotopically enriched NO_3_^-^ that had leached into the streams post-fire. A single measurement of higher streamwater NO_3_^—^δ^15^N from the streams of three burned watersheds relative to three streams of paired unburned watersheds is consistent with this hypothesis. Post-fire increases in streamwater NH_4_^+^ concentrations, however, have been reported for the first post-fire year after a wildfire [57]. Although we did not observe any difference in NH_4_^+^ concentrations between paired watersheds, we cannot be certain about isotopic enrichment. Uptake of leached and ^15^N-enriched NH_4_^+^ by moss might therefore have contributed to higher moss δ^15^N observed in burned watersheds. Alternatively, rather than reflecting a terrestrial isotope signal, altered in-stream N cycling (due to likely higher light availability and water temperatures after fire) could have also contributed to increased moss δ^15^N and deserves further study. However, if the post-fire increased moss δ^15^N largely reflected a terrestrial isotope signal, it would highlight the importance of terrestrial N inputs for aquatic productivity in N limited streams. It would further demonstrate retention of part of the leached terrestrial N by stream biota and support subsequent stream-internal N cycling and reciprocal exchanges with the land via stream water N uptake by riparian plants [56] or via interdependent food webs [58,59]. Terrestrial N was not exported into streams after spring prescribed burns as was reflected in the lack of an isotopic fire signal in moss and corroborated by streamwater NO_3_^-^ concentrations that were similar to those of unburned watersheds [6].

In wildfire-burned watersheds, the similarity in isotopic response between riparian foliage and in-stream moss is intriguing. Since we sampled moss in late May/early June and, coincidentally, post-fire year 1 riparian foliage at the same time, we hypothesize that abundant, post-fire flushed soil N (as indicated by higher moss N concentrations in burned vs unburned watersheds) had mainly a pre-fire isotopic signature during the first spring after wildfire and that isotopic shifts became pronounced over the course of the first post-fire growing season. This is supported by the presence of distinct post-fire isotopic enrichment in upland foliage in July/August of post-fire year 1 and in moss and riparian plants in subsequent post-fire years.

## Conclusions

Our study of post-fire N cycling showed a tight coupling among terrestrial, riparian, and aquatic systems in small, N-limited watersheds in the Rocky Mountains. Specifically, this is one of the first studies to demonstrate that streamwater and in-stream non-vascular plants can possibly reflect changes in soil isotopic signature due to wildfire. An isotopic fire signal from upland areas to aquatic autotrophs can then potentially be traced through the food web. However, a possible role of altered in-stream N cycling post-fire and its effects on aquatic δ^15^N needs to be further investigated. Riparian areas, the interface between terrestrial and aquatic ecosystem components, integrated local N cycling, distinct from upland areas, and intercepted N leached from upland areas. Riparian plant foliage showed an isotopic fire signal while a concomitant increase in foliar N concentration was not observed. The former highlights the riparian role of retaining N potentially leached from upland areas and emphasizes the need to study riparian N transformation processes when investigating watershed-level N cycling. At prescribed burn sites, our isotope data of in-stream moss support our conclusion in Stephan et al. [6] that low-severity spring prescribed burns do not provide the stream ecosystem with potentially important nutrient pulses. In the terrestrial component of watershed ecosystems, N isotope data of upland foliage provided more conclusive evidence than N concentration data alone, for a stronger alteration of N cycling after wildfire than after spring prescribed burns.

The major difference between our isotopic and N concentration results was that fire effects on N concentrations in terrestrial components (soil, upland foliage) appeared to decrease quickly from post-fire year 1 to post-fire year 3, while isotopic enrichment did not. This is relevant when choosing indicators for the duration of fire effects on ecosystems. We therefore suggest that the isotopic signal in deciduous foliage appears to be a better indicator of the duration of altered terrestrial N cycling than readily measured soil inorganic or foliar N concentrations. The utility of N isotopes for studying fire effects on N cycling would be greatly enhanced by the development of inexpensive, reliable methods for measuring isotopic values of dissolved inorganic nitrogen in soil and streamwater.

## Supporting Information

S1 Dataset Raw data. (XLS)
